# Urinary metabolite signature in bipolar disorder patients during depressive episode

**DOI:** 10.18632/aging.101805

**Published:** 2019-02-05

**Authors:** Jian-jun Chen, Jing Xie, Li Zeng, Chan-juan Zhou, Peng Zheng, Peng Xie

**Affiliations:** ^1^Institute of Life Sciences, Chongqing Medical University, Chongqing, China; ^2^Department of Neurology, The First Affiliated Hospital of Chongqing Medical University, Chongqing, China; ^3^Institute of Neuroscience, Chongqing Medical University, Chongqing, China; ^4^Department of Nephrology, The First Affiliated Hospital of Chongqing Medical University, Chongqing, China; ^5^Department of Endocrinology and Nephrology, Chongqing University Central Hospital, Chongqing Emergency Medical Center, Chongqing, China; ^*^Equal contribution

**Keywords:** bipolar disorder, metabolites, metabolomics, biomarker

## Abstract

The first few episodes of bipolar disorder (BD) are highly likely to be depressive. This phenomenon causes many BD patients to be misdiagnosed as having major depression. Therefore, it is very important to correctly diagnose BD patients during depressive episode. Here, we conducted this study to identify potential biomarkers for young and middle-aged BD patients during depressive episode. Both gas chromatography-mass spectroscopy (GC-MS) and nuclear magnetic resonance (NMR) spectroscopy were used to profile the urine samples from the recruited subjects. In total, 13 differential metabolites responsible for the discrimination between healthy controls (HCs) and patients were identified. Most differential metabolites had a close relationship with energy homeostasis. Meanwhile, a panel consisting of five differential metabolites was identified. This panel could effectively distinguish the patients from HCs with an AUC of 0.998 in the training set and 0.974 in the testing set. Our findings on one hand could be helpful in developing an objective diagnostic method for young and middle-aged BD patients during depressive episode; on the other hand could provide critical insight into the pathological mechanism of BD and the biological mechanisms responsible for the transformation of different episodes.

## INTRODUCTION

Bipolar disorder (BD) is a debilitating mental disorder, which could cause periods of depression, mania and euthymia [1]. It is the 6th leading cause of disability worldwide, and its lifetime prevalence is about 33% in the general population [2, 3]. However, the exact pathogenesis of BD is still poorly understood. The gene study showed that the genetic heritability could increase the predisposition of BD [4]. Up to now, there is still lack of objective diagnostic methods for BD, which makes the diagnosis only rely on the subjective identification of symptomatic clusters [5]. But, the clinical symptoms of this disease are considerably complex and diverse. Then, the current symptom-based method results in a considerable error rate [6]. An approach to circumvent these limitations is to find diagnostic biomarkers for BD.

Recently, many works have been done to identify diagnostic biomarkers for BD [7–11]. Lan et al. found several significantly altered metabolites in the brain tissue of BD patients [7]. Another study found that the serum phosphatidylinositol might be a potential biomarker for BD liability [8]. Our previous studies have also identified some potential biomarkers for BD diagnosis [9, 10]. These findings could be helpful in developing objective diagnostic methods for BD. However, these previous studies have not taken the different episodes of BD into consideration. BD patients during different episodes might have different symptoms. Previous studies showed that there were attentional biases toward negatively valenced stimuli in manic and depressive BD patients [11, 12]. Cunha et al. found that the brain-derived neurotrophic factor level was significantly decreased in manic and depressed BD patients than in euthymic BD patients [13]. In addition, metabolomics is used to capture the metabolic alterations in various disease states. Patients in different disease states might have the different metabolic phenotypes. Moreover, using different treatment methods to treat BD patients during different episodes could be a good choice. A clinical review recommended that the antidepressants should be stopped in period of mania, and the antidepressants should be used with a mood stabilizer in period of depression [1]. Given these facts, there is an urgent need to develop objective diagnostic methods for BD patients during different episodes.

Currently, there are three major analytical techniques that could be used for non-targeted metabolomic mapping: liquid chromatography-mass spectroscopy (LC-MS), gas chromatography-mass spectroscopy (GC-MS) and nuclear magnetic resonance (NMR) spectroscopy. Each technique has its advocates and possesses their own unique features. But, no single technique could provide adequate coverage of the entire human metabolome in any given biological sample [14]. Previous studies have reported that the combined application of multiple techniques could substantially enhance the level of metabolome coverage and yield more meaningful results [15–17]. Therefore, in this study, a dual platform approach (GC-MS and NMR) was used to study the metabolic phenotype in young and middle-aged BD patients during depressive episode. The urine was used here, because it could be non-invasively collected in clinical practice, which was commendable and might have better clinical utility.

## RESULTS

### Discriminative model construction

**Figure 1:**
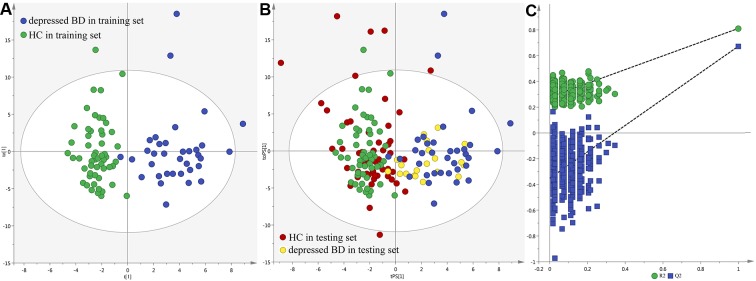
**Metabolomic analysis of urine samples from the recruited subjects.** (**A**) OPLS-DA model; (**B**) T-predicted scatter plot; (**C**) 300-iteration permutation test.

The score plot of the orthogonal partial least-squares discriminant analysis (OPLS-DA) model showed that the patients and healthy controls (HCs) could be clearly separated with little overlap (R2Y=0.81, Q2Y =0.67; Figure 1A). The positive values of R2Y and Q2Y showed that there was a robust metabolic difference between these two groups. This model could also correctly predict the samples from the testing set (Figure 1B). The T-predicted scatter plot showed that 51 of the 55 HCs and 19 of the 20 patients were correctly predicted. The average predictive accuracy was 93.3%. These results indicated that the OPLS-DA model built with urinary metabolites could be a potential tool for objectively diagnosing young and middle-aged BD patients during depressive episode. Moreover, the higher original Q2 and R2 values than their corresponding permutated values demonstrated that the built OPLS-DA model was valid and not over-fitted (Figure 1C), which further declared the robust of these results.

### Differential metabolites identification

**Figure 2:**
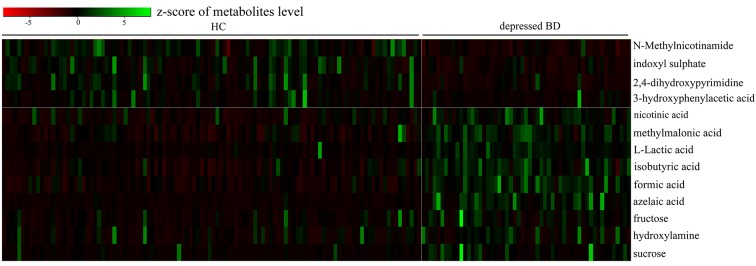
Heatmap of the 13 identified differential metabolites.

**Figure 3:**
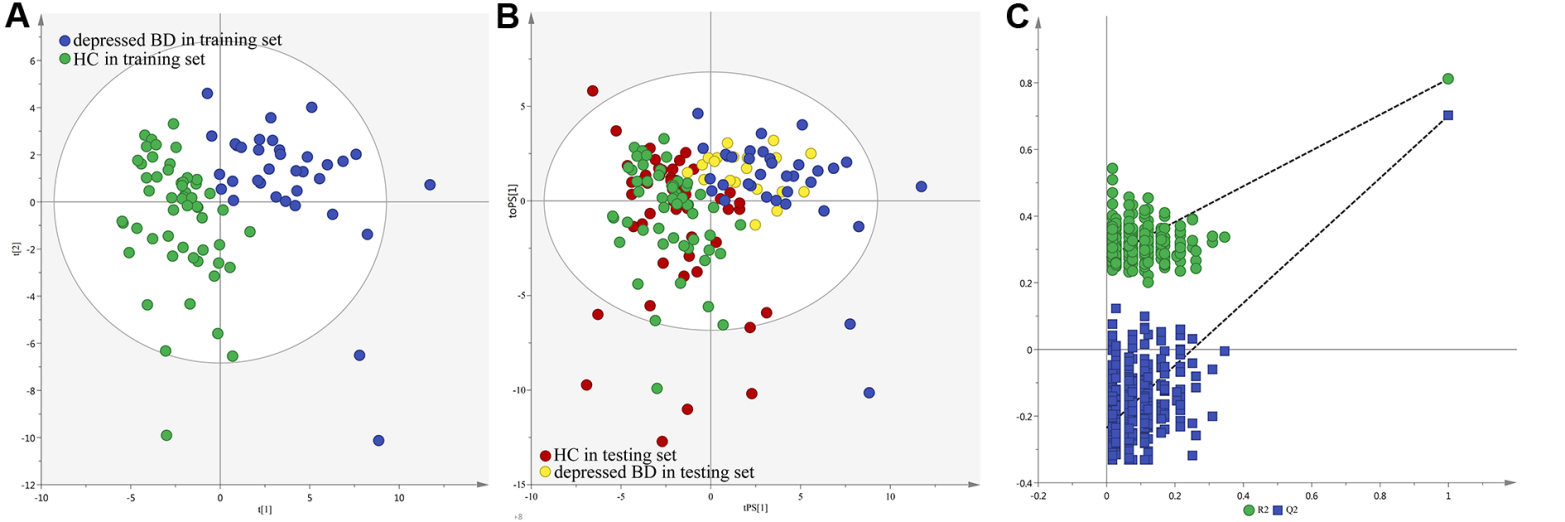
**Metabolomic analysis of urine samples from the recruited subjects.** (**A**) PLS-DA model; (**B**) T-predicted scatter plot; (**C**) 300-iteration permutation test.

The loading coefficient plot showed that there were 13 differential metabolites (|r|>0.430) responsible for the discrimination between HCs and patients (Figure 2). As compared to HCs, the patients were characterized by significantly higher levels of nicotinic acid, methylmalonic acid, L-Lactic acid, isobutyric acid, formic acid, azelaic acid, fructose, hydroxylamine and sucrose, along with significantly lower levels of N-Methylnicotinamide, indoxyl sulphate, 2,4-dihydroxypyrimidine and 3-hydroxyphenylacetic acid. Meanwhile, we used the non-parametric Mann-Whitney U test to further validate the metabolic changes identified by the OPLS-DA model, and found that these urinary metabolites levels remained significantly changed. The detailed information was described in Table 1. Meanwhile, we used the training set to build the PLS-DA model and the testing set to independently validate the built model. The results showed that these two groups could be clearly separated with little overlap (R2Y=0.81, Q2Y =0.70; Figure 3A and 3B). The 300-item permutation test showed that the built model was valid (Figure 3C). By analyzing the loading coefficient plot of the built PLS-DA model, we obtained the same differential metabolites (Table 1). These results showed that the OPLS-DA and PLS-DA could lead to the same metabolite signatures, further suggesting the robust of our results.

**Table 1:** Differential metabolites responsible for the discrimination of two groups

Metabolite	p-value^a^	p-value^b^	R^c^	R^d^	FC^e^	Metabolic classification
N-Methylnicotinamide	2.30E-10	1.25E-09	-0.94	-0.94	2.57	Metabolism of cofactors and vitamins
indoxyl sulphate	8.50E-06	2.73E-05	-0.61	-0.62	1.77	Gut microbial metabolites
2,4-dihydroxypyrimidine	6.31-08	2.74E-07	-0.5	-0.47	1.84	Carbohydrate metabolism
3-hydroxyphenylacetic acid	0.033	0.05	-0.55	-0.54	2.22	Amino acid metabolism
nicotinic acid	1.48E-07	5.88E-07	0.49	0.49	0.47	Metabolism of cofactors and vitamins
methylmalonic acid	8.46E-12	6.69E-11	0.48	0.47	0.62	Carbohydrate metabolism
L-Lactic acid	2.09E-18	1.81E-16	0.43	0.43-	0.44	Carbohydrate metabolism
isobutyric acid	5.85E-13	5.66E-12	0.56	0.56	0.37	Carbohydrate metabolism
formic acid	1.31E-14	2.27E-13	0.47	0.47	0.47	Energy metabolism
azelaic acid	8.23E-16	1.79E-14	0.62	0.61	0.12	Lipid metabolism
fructose	3.56E-06	1.19E-05	0.64	0.64	0.35	Carbohydrate metabolism
hydroxylamine	0.014	0.025	0.47	0.46	0.67	Energy metabolism
sucrose	4.72E-08	2.16E-07	0.78	0.78	0.20	Carbohydrate metabolism

^a^p-values were derived from non-parametric Mann-Whitney U test.

^b^adjusted p-values were derived from Benjamini and Hochberg False Discovery Rate.

^c^correlation coefficient obtained from the OPLS-DA model, positive and negative values indicated higher and lower levels in depressed BD subjects, respectively.

^d^correlation coefficient obtained from the PLS-DA model, positive and negative values indicated higher and lower levels in depressed BD subjects, respectively.

^e^FC, fold change; <1 and >1 indicated higher and lower levels in depressed BD subjects, respectively.

### Pearson correlation analysis

**Figure 4:**
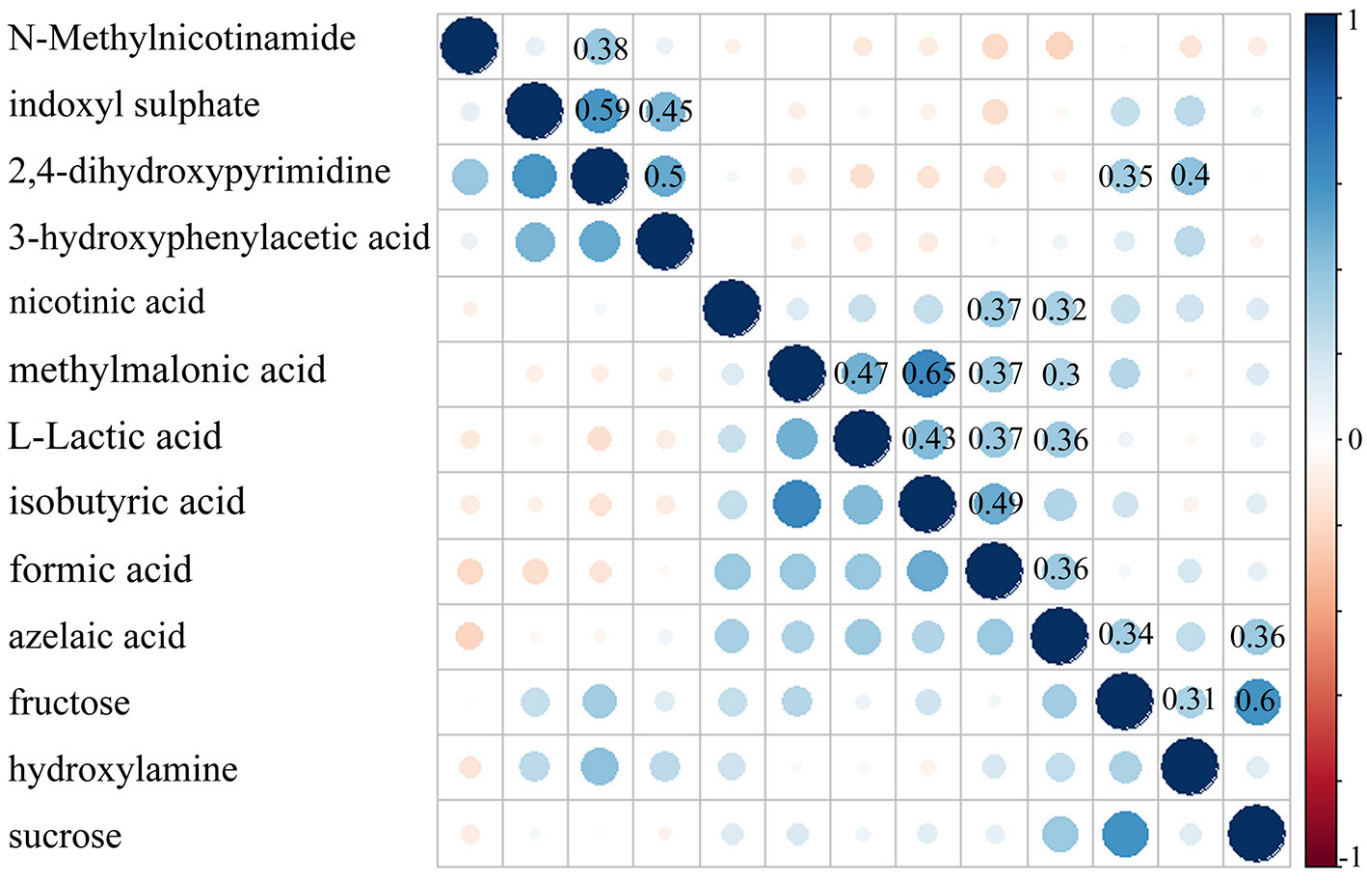
Pearson correlation coefficient of the 13 identified differential metabolites.

**Figure 5:**
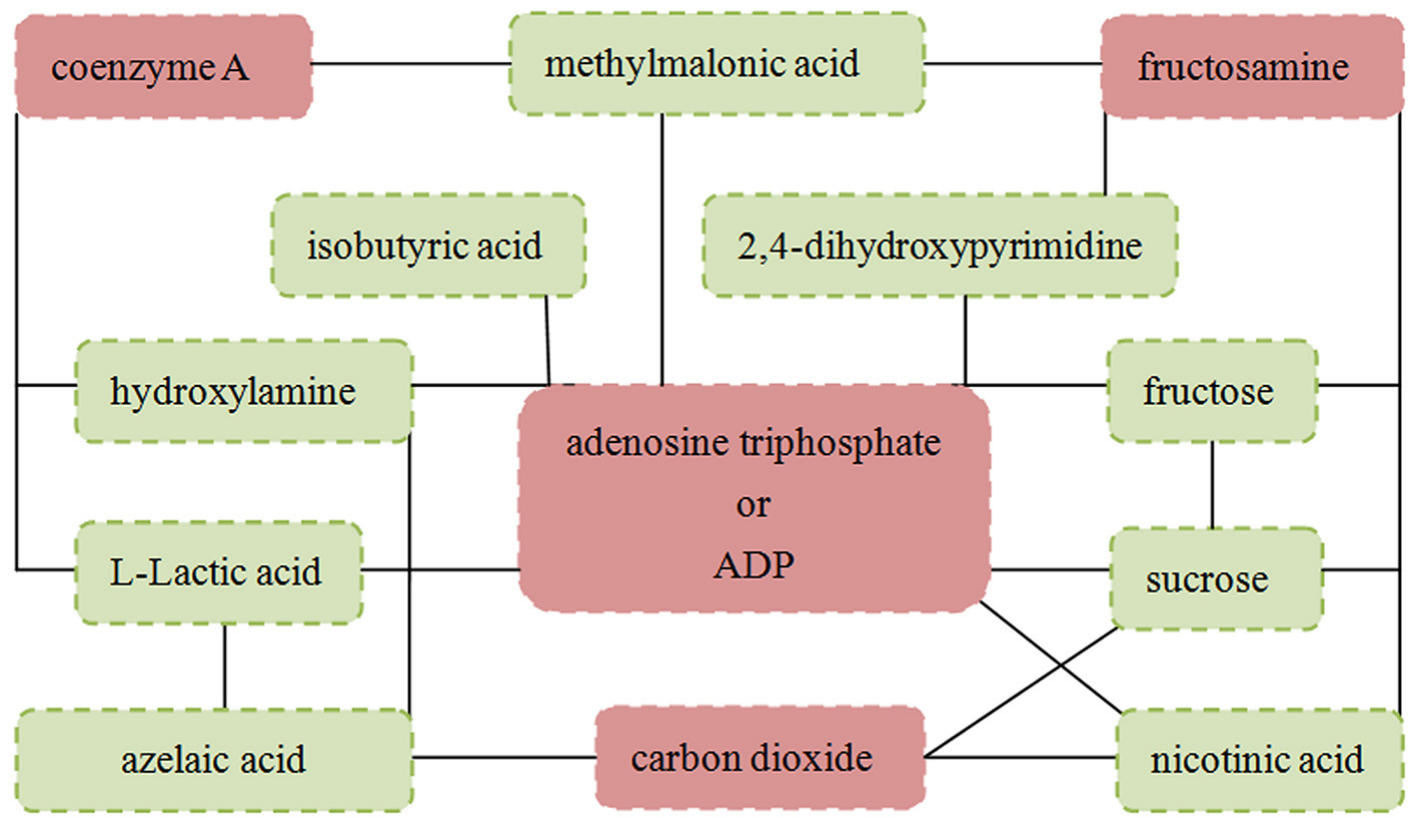
Metabolite-metabolite interaction network of the 13 identified differential metabolites.

The Pearson correlation analysis was conducted to show the relationship between the differential metabolites. As shown in Figure 4, we found that: i) the four significantly decreased metabolites were positively correlated with each other; ii) the six significantly increased metabolites (nicotinic acid, methylmalonic acid, L-Lactic acid, isobutyric acid, formic acid and azelaic acid) were positively correlated with each other; and iii) the other three significantly increased metabolites were positively correlated with each other. Meanwhile, the metabolite-metabolite interaction network showed that 9 of the 13 differential metabolites could interact with each other directly or through one metabolite, and most metabolites had a close relationship with carbohydrate metabolism and energy metabolism (Figure 5).

### Simplified panel identification

**Figure 6:**
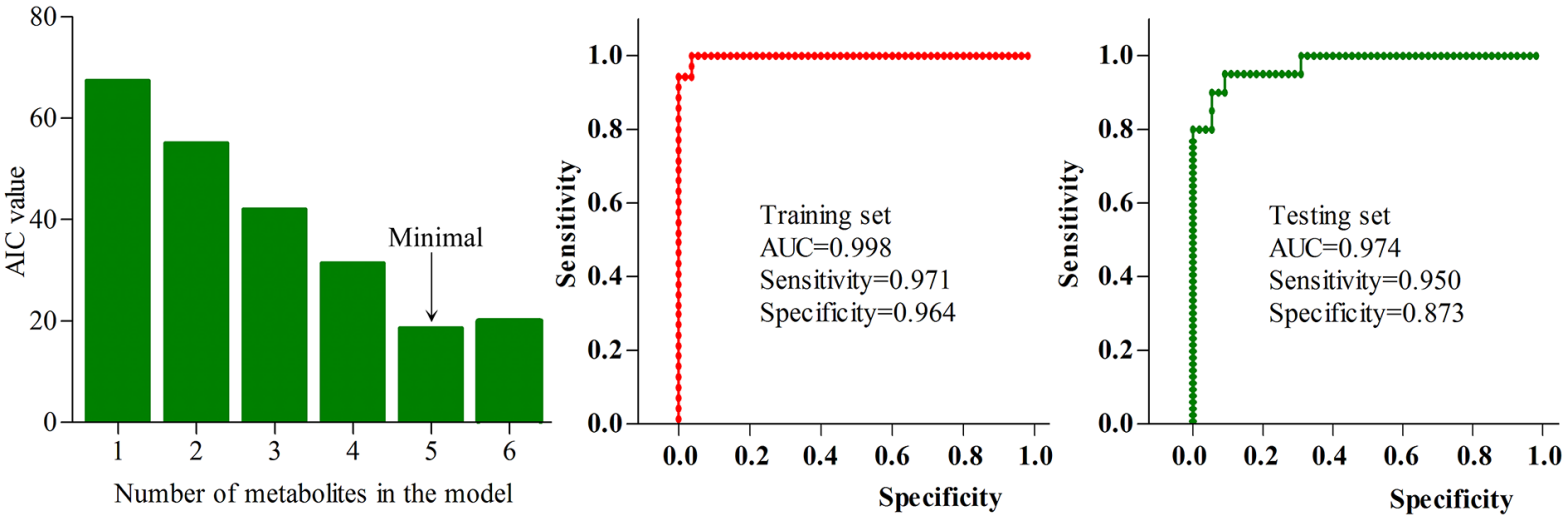
Diagnostic performance of the simplified biomarker panel.

Using logistic-regression analysis and Akaike’s information criterion (AIC) rule, we found that the most significant deviations between HCs and patients could be explained by five urinary metabolites: isobutyric acid, formic acid, 2,4-dihydroxypyrimidine, azelaic acid and sucrose (Figure 6). The panel consisting of these metabolites: P(Y=1)=1/(1+e-y); y=1/(1+EXP(-563.937*isobutyric acid- 4456.691*formic acid+405.422*2,4-dihydroxypyrimidine-1970.034*azelaic acid-58.149*sucrose+ 30.976) could be used to calculate the probability of illness in each sample. Meanwhile, we further used the receiver operating characteristic (ROC) curve analysis to assess the diagnostic performance of this simplified panel (Figure 6). The results showed that this panel could effectively distinguish the patients from HCs with an area under the ROC curve (AUC) of 0.998 in the training set (sensitivity=0.971, specificity=0.964) and 0.974 in the testing set (sensitivity=0.950, specificity=0.873). These results indicated that this panel could be a ‘good’ classifier of depressed BD patients and HCs.

### Effects of medication on metabolites

**Figure 7:**
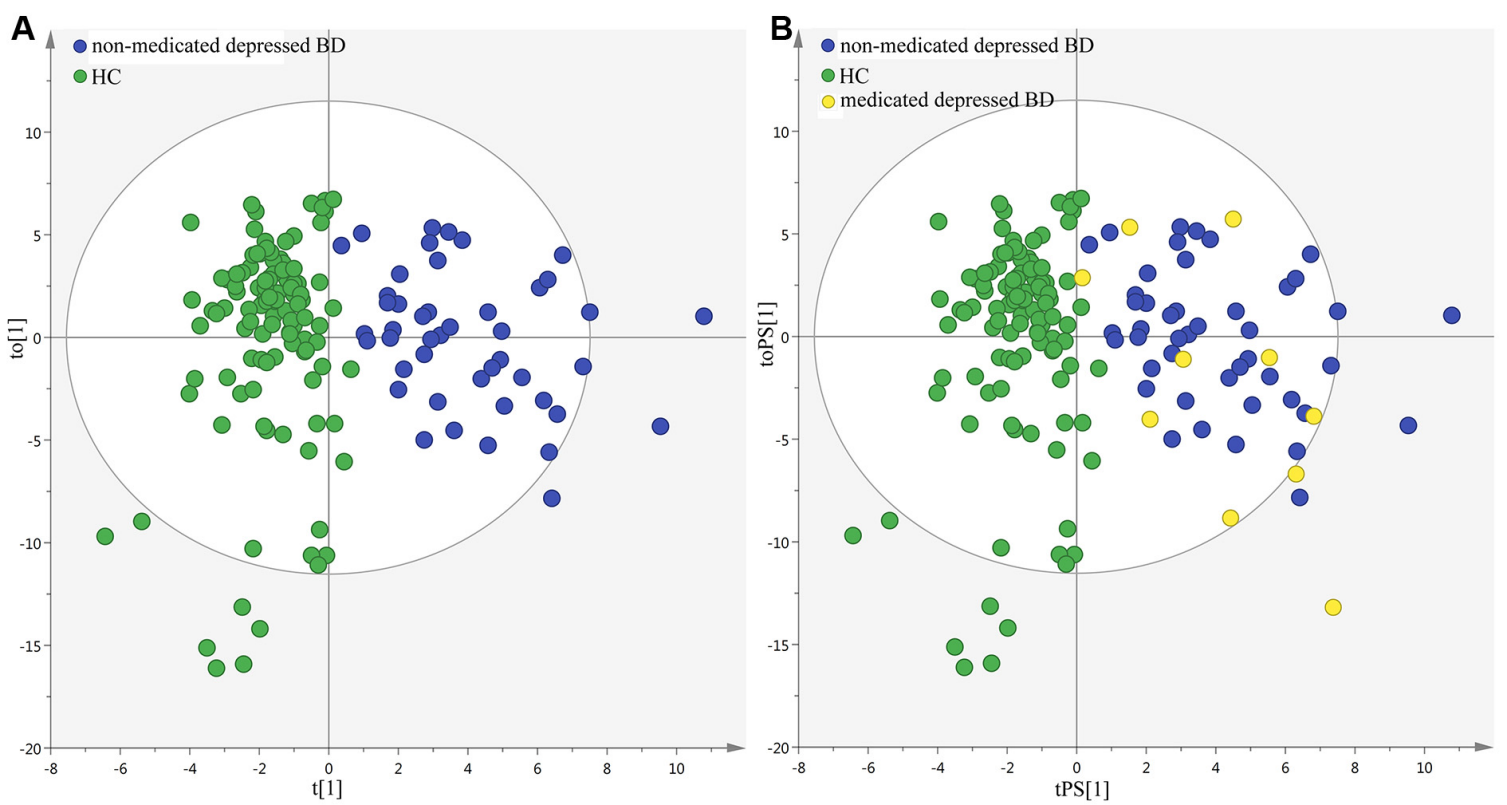
Metabolic phenotypes homogeneity between non-medicated and medicated patients.

There were ten medicated patients. To determinate the homogeneity of metabolic phenotypes between the medicated and non-medicated patients, we firstly built the OPLS-DA model using the non-medicated patients and HCs (Figure 7A). Then, the constructed OPLS-DA model was used to predict the class membership of the medicated patients. The results showed that the 10 medicated patients were correctly predicted by the built OPLS-DA model ((Figure 7B). These findings indicated that the metabolic phenotypes were significantly different between the non-medicated patients and HCs, but not between the medicated and non-medicated patients. Meanwhile, the simplified panel could effectively discriminate the medicated patients from HCs. Therefore, the medication might have little impact on metabolites in urine. Limited by the small sample size of medicated patients, this conclusion was needed future studies to validate.

## DISCUSSION

This was the first study to explore the metabolic changes in young and middle-aged BD patients during depressive episode. The multiple metabolomics differential metabolites responsible for the discrimination between the patients and HCs. Most differential metabolites were associated with carbohydrate metabolism and energy metabolism, which indicated that the energy homeostasis might be disturbed in depressed BD patients. Furthermore, a potential biomarker panel consisting of five differential metabolites was identified. This panel could correctly diagnose the patients with an AUC of 0.998 in the training set and 0.974 in the testing set. Our results could be helpful in developing an objective diagnostic method for depressed BD patients, and provide novel insight into the pathogenesis of BD.

Energy homeostasis is an important biological process, which is involved in the coordinated homeostatic regulation of food intake and energy expenditure [18, 19]. The human brain, particularly the hypothalamus, plays an important role in regulating the energy homeostasis [20, 21]. In recent decades, the hypothalamic-pituitary-adrenal (HPA) axis has become the focus of researches on investigating the pathogenesis of neuropsychiatric diseases [22]. The consequences of HPA dysfunction might be central to the pathogenesis of neuropsychiatric diseases. Fries et al. reported that a dysfunctional HPA axis might play a key role in the pathophysiology of illness progression in BD [23]. A previous study reported that the brain energy metabolism in BD patients was altered [24]. Our previous studies have also identified some differential urinary metabolites that were involved in energy metabolism in depressed patients [25, 26]. Meanwhile, we also observed the perturbed energy metabolism in the cerebellum of chronic mild stressed-treated depressed rats [27]. Here, we found that the energy homeostasis might be disturbed in depressed BD patients. Based on these results, we deduced that the pathogenesis of BD might be associated with the disturbance of energy homeostasis that was caused by the dysfunctional HPA axis.

Gut microbiota has a fundamental role in the well-being of their host [28–30]. It could influence many aspects of human physiology. The disturbed gut microbiota has been found to be closely related with many diseases, such as diabetes and obesity [31, 32]. Some studies have shown that it could influence the brain function through the microbiota-gut-brain axis [33, 34]. Our previous study found that the gut microbiota could increase or decrease the gene expression levels in the hippocampus of mice, and the differentially expressed gene were mainly related with carbohydrate metabolism [35]. Meanwhile, our clinical studies have proved that the gut microbiota might have a causal role in the development of depressive-like behaviors [36, 37]. In this study, we identified three differential metabolites (isobutyric acid, formic acid and indoxyl sulphate) belonging to the metabolic byproducts of gut microbiota. These results indicated that the dysbiosis of gut microbiota might have a causative role in the onset of BD.

Both mania and depression are characterized by the disturbance in cognition, circadian rhythm, normal mood and psychomotor activity. The biological mechanism responsible for switching from depressive episode to manic episode, or vice versa, is still poorly understood [38]. BD patients during manic episode generally exhibit some abnormal behaviors, such as uninterruptible manner, speaking in a rapid, short attention span, decreased need for sleep [39]. If untreated, the manic episode could last three to six months. BD patients during depressive episode also have some abnormal behaviors, such as loss of interest in previously enjoyed activities, feelings of worthlessness, inappropriate guilt, sadness, hopelessness, and thoughts of death or suicide [40]. If untreated, the depressive episode could last at least two weeks, and may result in suicide. In clinical practice, the first few episodes of BD are highly likely to be depressive [41, 42]. This phenomenon causes many BD patients to be misdiagnosed as having major depression and then improperly treated with antidepressants [43]. Our present findings could facilitate the development of an objective diagnostic method for depressed BD patients, and provide critical insight into the biological mechanism responsible for the transformation of different episodes.

Several limitations should be mentioned here. Firstly, only 55 patients were included in this study; then our conclusions were still needed future studies to verify and support. Secondly, all subjects came from the same place, which might limit the applicability of our conclusion [44]. Thirdly, only the metabolic phenotype of BD patients during depressive episode was explored here, future studies should further study the metabolic phenotype of BD patients during other episodes. Fourthly, although our results showed the similar metabolic phenotype between the medicated and non-medicated patients, future studies were still needed to assess the effects of medication on urinary metabolites. Finally, only urine sample was used here, future studies should collect other biosamples, such as cerebrospinal fluid (CSF), to ensure whether these differential urinary metabolites were physiologically relevant to the disease pathogenesis.

In conclusion, using the dual platform approach (GC-MS and NMR), we identified 13 differential metabolites in the urine of young and middle-aged BD patients during depressive episode. These differential metabolites indicated that the energy homeostasis might be disturbed in depressed BD patients. Meanwhile, a panel consisting of five differential metabolites that could effectively distinguish the patients from HCs was identified. Our results could lay the groundwork for future developing an objective method for diagnosing BD patients during depressive episode, and studying the pathogenesis of BD.

## MATERIALS AND METHODS

### Subject Recruitment

The protocol of our study was approved by the Ethical Committee of Chongqing Medical University and all subjects provided written informed consent. In total, 55 young and middle-aged BD patients during depressive episode were recruited from the First Affiliated Hospital of Chongqing Medical University. The diagnosis was conducted based on the Structured Clinical Interview from the DSM-IV-TR. The depressive and manic symptoms were assessed using the 17-item Hamilton Depression Rating Scale (HDRS) and 11-item Bech-Rafaelsdn Mania Rating Scale (BRMS), respectively. The BD patients were considered to be in depressive episode, if they scored >17 on HDRS scale and <6 on BRMS scale. The patients with any pre-existing physical or other mental disorders were excluded. The outpatients were recruited, and there were ten medicated patients. Meanwhile, 110 sex-, age- and body mass index (BMI)-matched HCs were recruited from the medical examination center of the First Affiliated Hospital, and they were required to have no current or previous lifetime history of major psychiatric disorders, dementia, cancer, and/or systemic medical illness. The detailed information of the included subjects was described in Table 2.

**Table 2:** Demographic and clinical details of recruited subjects

Variables	Patients	HCs	p-value
Sample Size	55	110	-
Medication(Yes/No)	10/45	0/110	p<0.00001^a^
Sex (Male/Female)	31/24	70/40	0.36^a^
Age (year)	31.76(11.44)	32.47(10.64)	0.69^b^
BMI	21.85(2.40)	21.53(2.78)	0.47^b^
HDRS	22.87(4.22)	1.36(1.22)	p<0.00001^b^
BRMS	3.24(0.99)	1.33(1.08)	p<0.00001^b^

HCs, healthy controls; BMI, body mass index; HDRS, Hamilton Depression Rating Scale; BRMS, Hamilton Anxiety Rating Scale; BRMS: Bech-Rafaelsdn Mania Rating Scale.

^a^p-value obtained from Chi-square analyses; ^b^p-value obtained from Two-tailed student T test.

### Data obtaining

It was critical to use the testing set to independently validate the results obtained from the training set. Therefore, the recruited subjects were randomly divided into two sets: training set and testing set. Finally, there were 55 HCs and 35 patients in the training set, and 55 HCs and 20 patients in the testing set. After overnight fasting, the morning (9:00-10:00 am) midstream urine samples of the included subjects in both sets were collected and then centrifuged at normal temperature (1500 g x 10 minutes). Then, we equally divided the obtained supernatant and stored them at -80°C for later analysis.

### NMR and GC-MS procedure

The procedure of NMR and GC-MS were performed exactly according to our previous studies [9, 10]. Briefly, for NMR analysis: 1) thawed and centrifuged (1500g for 10 minutes) each sample to remove precipitation; 2) mixed each sample (500µl) with phosphate buffer (90% D2O, 1 mM 3-trimethylsilyl-1-[2, 2, 3, 3-²H4] propionate (TSP), and 3 mM sodium azide; pH 7.4) (100µl) to ensure the stabilization of urinary pH; 3) after centrifugation (12000rpm for 10 minutes), transferred the supernatant (500 µl) into 5mm NMR tubes; 4) collected the proton spectra of each sample using Bruker Avance 600 spectrometer operating at a 600.13 MHz 1H frequency with a standard 1-dimensional (1D) pulse sequence (see the specific parameters in our previous study [9]). For GC-MS analysis: 1) vortexed the urine sample (15µl) and internal standard solutions (L-leucine-13C6, 0.02 mg/ml) (10µl); 2) added urease (15µl) into the mixture solution to degrade the urea (one hour at 37°C); 3) extracted the mixture solution successively using 240μl and 80μl ice-cold methanol, and then vortexed for 30 seconds; 4) after centrifugation (14000rpm for 5 minutes at 4°C), transferred the supernatant (224 µl) into a glass vial and then conducted vacuum-drying at room temperature; 5) derivatized the dried metabolic extract using methoxyamine (20 mg/ml) (30µl) (1.5 hours at 37°C); 6) added BSTFA (with 1% TCMS) (30µl) into the mixture, and then heated it (one hour at 70°C) to form trimethylsilyl (TMS) derivatives; 7) after cooling to room temperature, injected the obtained derivative (1.0µl) into the GC-MS system (see the specific parameters in our previous study [10]).

### Statistical analysis

In order to alleviate the effects of the different samples, we used the creatinine to normalize the original spectral data of metabolites. Then, in order to eliminate the effects of different orders of magnitude, the obtained data was scaled to zero-mean and unit-variance. Finally, the processed data was imported into SIMCAP+ 14.0 software to build OPLS-DA model. This model was used to visualize the discrimination between the different groups. The R2X, R2Y and Q2Y were viewed as the evaluation indexes of the quality of OPLS-DA model [45, 46]. Meanwhile, we conducted 300-iteration permutation test to assess whether or not there was non-randomness of separation between the different groups.

By analyzing the coefficient loading plot of OPLS-DA model, we could obtain the differential metabolites responsible for samples separation [47]. According to the number of samples using to build OPLS-DA model, we selected a correlation coefficient of |r|>0.430 (equivalent to a p-value<0.01) as cut-off value. Then, we used correlation analysis to assess the correlations between the identified differential metabolites. Meanwhile, to study the interaction of these differential metabolites, we used the online software MetaboAnalyst 3.0 to build metabolite-metabolite interaction network. Also, the nonparametric Mann-Whitney U test and Benjamini-Hochberg False Discovery Rate were used to check whether these differential metabolites were still significantly changed or not between the different groups.

In clinical practice, it would be more convenient and feasible to make a diagnosis based on several metabolites. Thus, in order to find a simplified biomarker panel, we used step-wise logistic-regression analysis (based on AIC rule) to further analyze these differential metabolites. The AIC estimated the quality of each model for a given set of data. It dealt with the trade-off between the simplicity and the goodness of fit of the model. Thus, it could be used to perform model selection. The model with the minimum AIC value was the preferred model. To assess the diagnostic performance of this simplified panel, we conducted ROC curve analysis. The AUC was the evaluation index. The accuracy of classification of this panel was excellent if the AUC value was between 0.9 and 1 [48].
